# Supplementary material for: ‘Dr. Google, What Is That on My Skin?’—Internet Searches Related to Skin Problems: Google Trends Data from 2004 to 2019
Source: Int J Environ Res Public Health. 2021 Mar 4;18(5):2541. doi: 10.3390/ijerph18052541 (PMC7967401; doi:10.3390/ijerph18052541)

Table S1.
Checklist for Documentation of Google Trends research. Modified from Nuti et al.

**Section/Topic**	**Checklist item**
**Search Variables**	
Access Date	11 February 2021
Time Period	From January 2004 to 31 December 2019.
Query Category	All query categories were used
Region	Worldwide
Countries with Low Search Volume	Excluded
**Search Input**	
**Non-adjusted**	„Abrasion”, „Blister”, „Cafe au lait spots”, „Cellulite”, „Comedo”, „Dandruff”, „Eczema”, „Erythema”, „Eschar”, „Freckle”, „Hair loss”, „Hair loss pattern”, „Hiperpigmentation”, „Hives”, „Itch”, „Liver spots”, „Melanocytic nevus”, „Melasma”, „Nevus”, „Nodule”, „Papilloma”, „Papule”, „Perspiration”, „Petechia”, „Pustule”, „Scar”, „Skin fissure”, „Skin rash”, „Skin tag”, „Skin ulcer”, „Stretch marks”, „Telangiectasia”, „Vesicle”, „Wart”, „Xeroderma”
**Adjusted**	Topics: "Scar" + „Abrasion” / „Blister” / „Cafe au lait spots” / „Cellulite” / „Comedo” / „Dandruff” / „Eczema” / „Erythema” / „Eschar” / „Freckle” / „Hair loss” / „Hair loss pattern” / „Hiperpigmentation” / „Hives” / „Itch” / „Liver spots” / „Melanocytic nevus” / „Melasma” / „Nevus” / „Nodule” / „Papilloma” / „Papule” / „Perspiration” / „Petechia” / „Pustule” / „Skin fissure” / „Skin rash” / „Skin tag” / „Skin ulcer” / „Stretch marks” / „Telangiectasia” / „Vesicle” / „Wart” / „Xeroderma”
**Rationale for Search Strategy**	
For Search Input	The searched topics are related to dermatologic complaints. Because Google Trends enables to compare only five inputs at once we compared relative search volume of all topics with topic „Scar” (adjusted data). Therefore, we were able to compare all 35 topics in further analysis.
For Setting Chosen	We chose the all categories to not limit the output. We excluded countries with low search volume which may be sensitive for outliers

Table S2
Use of data generated by Google Trends.

	Interest over time	Region
		Interest by region
Non-adjusted data	Time series analysis: yearly trends, seasonal variation (Table 2, Figure 3)	Countries with the highest RSV of analyzed topics (Supplementary Table 4)
		Compared breakdown by region
Adjusted data	Proportion of mean RSV to topic „Scar” (Table 1)	List of the most common topics in a specific country (Figures 1-2, Supplementary Table 3)

RSV – Relative Search Volume

Figure S1

Data collection and data processing flowchart.

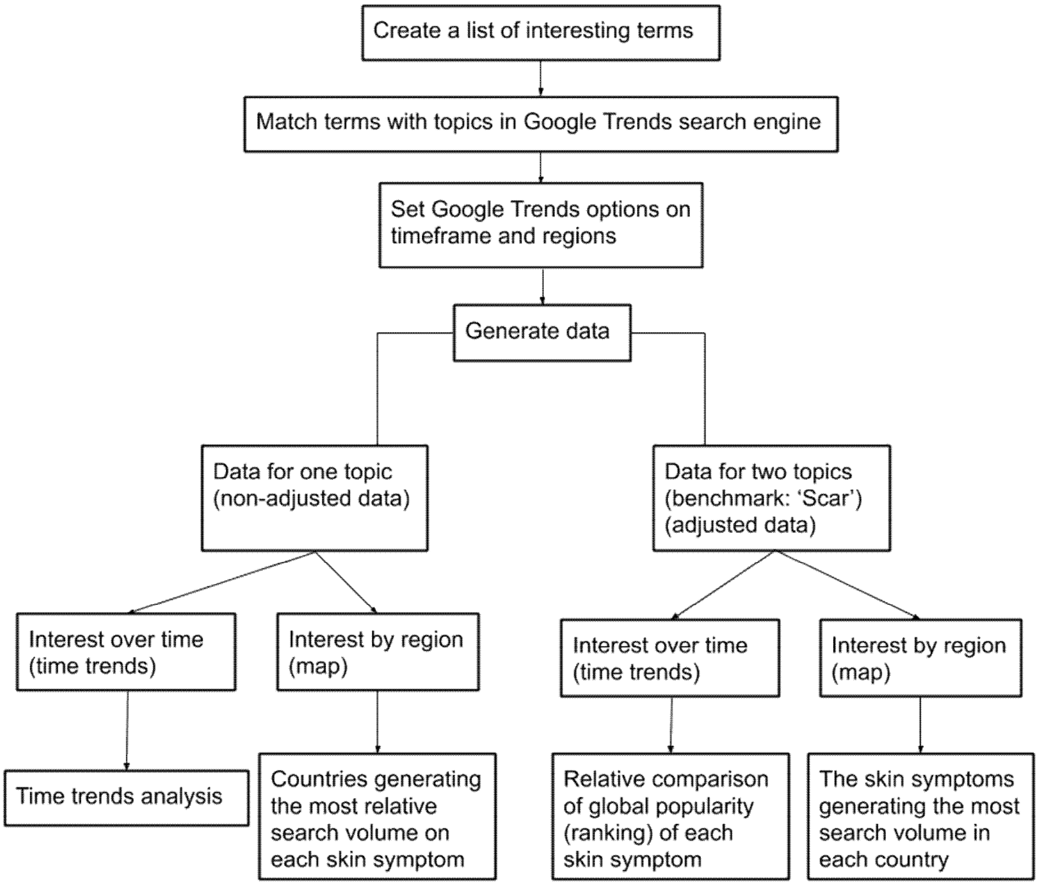

Table S3.
The five most common topics in a specific country.
Relative search volume estimated by using adjusted data of compared breakdown by region (Scar + another topic).

Country	The most common topic
Algeria	Itch (77), Hair loss (75), Perspiration (70), Dandruff (57), Comedo (55)
Argentina	Itch (66), Blister (57), Wart (57), Skin ulcer (52), Hair loss (51)
Australia	Itch (70), Skin rash (64), Hair loss (53), Perspiration (53), Scar (50)
Austria	Itch (64), Hair loss (63), Perspiration (60), Skin rash (58), Wart (50)
Belarus	Nevus (65), Skin rash (61), Wart (57), Papilloma (55), Cellulite (50)
Belgium	Itch (68), Perspiration (57), Hair loss (53), Skin rash (51), Wart (50)
Bolivia	Hair loss (54), Scar (50), Wart (45), Papilloma (40), Stretch marks (40)
Brazil	Itch (65), Nodule (61), Stretch marks (55), Nevus (54), Comedo (51)
Bulgaria	Hair loss (65), Itch (63), Perspiration (58), Skin rash (57), Scar (50)
Canada	Itch (67), Skin rash (61), Hair loss (56), Perspiration (52), Scar (50)
Chile	Itch (67), Papilloma (56), Hair loss (54), Stretch marks (51), Wart (50)
China	Itch (75), Perspiration (66), Eczema (54), Nevus (51), Hair loss (50)
Colombia	Papilloma (59), Hair loss (54), Itch (50), Stretch marks (50), Scar (50)
Croatia	Itch (74), Wart (64), Skin rash (63), Perspiration (60), Erythema (59)
Czechia	Itch (63), Skin rash (58), Wart (58), Perspiration (52), Scar (50)
Denmark	Itch (71), Skin rash (65), Perspiration (59), Eczema (57), Hives (53)
Ecuador	Papilloma (64), Itch (62), Hair loss (52), Scar (50), Perspiration (49)
Egypt	Hair loss (84), Perspiration (78), Dandruff (74), Itch (73), Wart (52)
Finland	Skin rash (83), Itch (79), Nevus (74), Perspiration (71), Papule (69)
France	Itch (58), Perspiration (57), Scar (50), Cellulite (49), Comedo (47)
Germany	Hair loss (66), Itch (66), Perspiration (63), Skin rash (56), Wart (54)
Greece	Itch (85), Wart (82), Cellulite (81), Hair loss (73), Eczema (66)
Hong Kong	Itch (68), Perspiration (65), Eczema (64), Hair loss (54), Skin rash (51)
Hungary	Itch (84), Hair loss (77), Wart (76), Eczema (74), Perspiration (69)
India	Hair loss (74), Itch (70), Perspiration (54), Dandruff (51), Scar (50)
Indonesia	Itch (83), Nodule (62), Comedo (59), Hair loss (56), Scar (50)
Ireland	Itch (75), Skin rash (70), Hair loss (60), Perspiration (57), Scar (50)
Israel	Itch (70), Skin rash (63), Hair loss (60), Perspiration (56), Scar (50)
Italy	Itch (74), Cellulite (59), Perspiration (57), Scar (50), Wart (49)
Japan	Itch (94), Perspiration (94), Hair loss (90), Nevus (90), Hives (87)
Kazakhstan	Itch (67), Skin rash (59), Nevus (55), Scar (50), Perspiration (48)
Kenya	Itch (83), Skin rash (71), Perspiration (65), Scar (50), Skin ulcer (43)
Malaysia	Itch (65), Scar (50), Comedo (40), Skin rash (36), Hair loss (33)
Mexico	Itch (71), Papilloma (70), Wart (57), Hair loss (54), Stretch marks (54)
Morocco	Itch (74), Hair loss (72), Perspiration (63), Dandruff (56), Scar (50)
Netherlands	Itch (77), Perspiration (60), Eczema (57), Hair loss (57), Skin rash (57)
New Zealand	Itch (73), Skin rash (67), Perspiration (55), Hair loss (54), Scar (50)
Nigeria	Itch (81), Skin rash (73), Stretch marks (61), Perspiration (59), Hair loss (51)
Norway	Skin rash (77), Itch (75), Perspiration (63), Nevus (57), Wart (53)
Pakistan	Hair loss (65), Itch (54), Scar (50), Perspiration (46), Skin rash (44)
Peru	Itch (60), Blister (52), Hair loss (52), Papilloma (52), Scar (50)
Philippines	Itch (55), Skin rash (50), Scar (50), Hair loss (35), Perspiration (34)
Poland	Wart (56), Itch (54), Skin rash (54), Scar (50), Pustule (49)
Portugal	Itch (68), Cellulite (61), Nodule (60), Hair loss (57), Blister (56)
Romania	Itch (81), Hair loss (77), Perspiration (73), Nodule (69), Cellulite (63)
Russia	Itch (61), Nevus (61), Skin rash (57), Papilloma (51), Scar (50)

Saudi Arabia	Itch (86), Perspiration (82), Hair loss (81), Dandruff (71), Eczema (63)
Serbia	Itch (74), Wart (62), Hair loss (60), Erythema (57), Cellulite (56)
Singapore	Itch (64), Skin rash (54), Hair loss (53), Scar (50), Perspiration (47)
Slovakia	Itch (77), Skin rash (77), Wart (65), Eczema (60), Scar (50)
South Africa	Itch (75), Skin rash (65), Perspiration (56), Hair loss (54), Skin ulcer (50)
South Korea	Hair loss (69), Perspiration (54), Itch (52), Scar (50), Wart (42)
Spain	Itch (72), Hair loss (55), Perspiration (52), Wart (52), Blister (51)
Sweden	Skin rash (71), Itch (65), Blister (63), Perspiration (60), Nodule (58)
Switzerland	Itch (63), Hair loss (61), Perspiration (59), Skin rash (52), Wart (50)
Taiwan	Itch (73), Perspiration (62), Skin rash (56), Nevus (53), Scar (50)
Thailand	Melasma (77), Skin ulcer (70), Freckle (65), Itch (65), Skin rash (60)
Turkey	Itch (96), Hair loss (94), Eczema (92), Perspiration (88), Wart (84)
Ukraine	Skin rash (66), Nevus (61), Itch (60), Papilloma (54), Wart (53)
United Arab Emirates	Itch (68), Hair loss (65), Perspiration (53), Skin rash (52), Scar (50)
United Kingdom	Itch (74), Skin rash (67), Hair loss (59), Perspiration (57), Scar (50)
United States	Itch (71), Skin rash (66), Hair loss (57), Perspiration (56), Scar (50)
Venezuela	Itch (67), Papilloma (67), Hair loss (63), Stretch marks (60), Blister (55)
Vietnam	Itch (83), Melanocytic nevus (74), Perspiration (62), Hair loss (57), Melasma (55)

Table S4
Five countries with the highest non-adjusted RSV by region of all topics representing dermatologic complaints.

Topic	Top five countries with highest RSV
Abrasion	Taiwan (100), Japan (73), Iran (72), Singapore (71), Philippines (64)
Blister	Taiwan (100), Sweden (96), Hong Kong (77), Ireland (70), United States (65)
Café au lait spot	Honduras (100), Chile (88), Guatemala (63), Costa Rica (59), Taiwan (48)
Cellulite	Greece (100), Portugal (93), Cyprus (92), Italy (92), Serbia (87)
Comedo	Indonesia (100), Malaysia (84), Thailand (66), Brazil (63), Hong Kong (55)
Dandruff	Sudan (100), Libya (67), Oman (58), Saudi Arabia (50), Kuwait (48)
Eczema	Hong Kong (100), China (57), Netherlands (49), Sweden (49), Hungary (47)
Erythema	Montenegro (100), Bosnia & Herzegovina (87), Croatia (71), Serbia (71), North Macedonia (44)
Eschar	Turkmenistan (100), Kazakhstan (69), Kyrgyzstan (52), Uzbekistan (38), Russia (33)
Freckle	Thailand (100), Vietnam (34), Iran (19), Jordan (15), Australia (13)
Hair loss	Iran (100), Singapore (56), United Arab Emirates (55), South Korea (53), United States (52)
Hives	Japan (100), Indonesia (83), Taiwan (72), Singapore (62), Iran (55)
Hyperpigmentation	Trinidad & Tobago (100), Mauritius (94), Singapore (87), South Africa (84), Nepal (64)
Itch	Indonesia (100), Iran (79), Vietnam (69), Taiwan (65), Malaysia (61)
Liver spot	Réunion (100), Jamaica (97), Guadeloupe (77), Madagascar (77), Tunisia (68)
Melanocytic nevus	Vietnam (100), Thailand (41), Singapore (27), United Kingdom (22), Bulgaria (21)
Melasma	Thailand (100), Laos (53), Libya (27), Vietnam (26), Japan (21)
Nevus	Taiwan (100), Iran (87), Japan (87), Brazil (71), Belarus (70)
Nodule	Indonesia (100), Brazil (79), Thailand (66), Sweden (49), Portugal (43)
Papilloma	Dominican Republic (100), Nicaragua (98), Panama (84), Costa Rica (83), Honduras (66)
Papule	Finland (100), Poland (30), Dominican Republic (23), Thailand (22), Ecuador (20)
Perspiration	Japan (100), Iran (90), Taiwan (86), Hong Kong (72), China (63)
Petechia	Paraguay (100), United States (95), Poland (90), Canada (85), Czechia (85)
Pustule	Poland (100), Slovenia (32), Germany (21), Austria (13), Italy (11)
Scar	Philippines (100), Malaysia (95), Singapore (78), Taiwan (70), Thailand (61)
Skin fissure	Japan (100), France (18), Réunion (13), Belgium (10), Vietnam (9)
Skin rash	Iran (100), Ireland (23), United States (23), Australia (22), United Kingdom (21)
Skin tag	United States (100), United Kingdom (95), Canada (92), Ireland (86), Australia (70)
Skin ulcer	Thailand (100), Puerto Rico (37), South Africa (33), Australia (27), Ghana (27)
Stretch marks	Brazil (100), Dominican Republic (97), Ghana (90), Honduras (87), Paraguay (85)
Telangiectasia	Brazil (100), Ukraine (71), Australia (61), Belarus (61), Ireland (61)
Vesicle	France (100), Poland (81), Algeria (65), Ecuador (59), Puerto Rico (59)
Wart	Bosnia & Herzegovina (100), Serbia (88), Croatia (86), Czechia (86), Poland (86)
Xeroderma	Ireland (100), United Kingdom (93), United States (91), Canada (82), Australia (80)

Table S5

Sensitivity analysis. Seasonal variation of interest in analyzed topics in three southern countries. The highlighted results represent inverse seasonal variation than that observed in the main analysis.

	**Australia**			**Brasil**			**South Africa**		
Topic	TBATS (seasonality present, period [month])	Month with the highest seasonal component [RSV]	Month with the lowest seasonal component [RSV]	TBATS (seasonality present, period [month])	Month with the highest seasonal component [RSV]	Month with the lowest seasonal component [RSV]	TBATS (seasonality present, period [month])	Month with the highest seasonal component [RSV]	Month with the lowest seasonal component [RSV]
Abrasion	NO, -	-	-	NO, -	-	-	NO, -	-	-
Blister	YES, 12	January (14.64)	June (-8.97)	YES, 12	January (6.56)	June (-3.15)	YES, 12	August (16.65)	May (-10.65)
Café au lait spot	NO, -	-	-	NO, -	-	-	NO, -	-	-
Cellulite	YES, 12	January (17.21)	June (-19.2)	YES, 12	November (10.11)	June (-10.44)	NO, -	-	-
Comedo	YES, 12	January (6.09)	May (-3.01)	YES, 12	January (3.53)	June (-2.56)	NO, -	-	-
Dandruff	YES, 12	June (7.37)	February (-7.63)	YES, 12	July (13.10)	March (-6.78)	NO, -	-	-
Eczema	NO, -	-	-	NO, -	-	-	NO, -	-	-
Erythema	YES, 12	July (5.87)	February (-4.49)	NO, -	-	-	NO, -	-	-
Eschar	NO, -	-	-	NO, -	-	-	NO, -	-	-
Freckle	YES, 12	January (10.49)	July (-9.29)	NO, -	-	-	NO, -	-	-
Hair loss	YES, 12	February (11.02)	August (-6.14)	YES, 12	April (12.25)	October (-9.66)	YES, 12	May (8.72)	December (-8.40)
Hives	YES, 12	January (8.00)	May (-8.89)	NO, -	-	-	NO, -	-	-

Hyperpigmentation	YES, 12	January (10.88)	June (-7.25)	NO, -	-	-	NO, -	-	-
Itch	YES, 12	January (6.93)	June (-3.76)	YES, 12	April (2.72)	November (-2.50)	YES, 12	December (3.00)	June (-4.61)
Liver spot	NO, -	-	-	NO, -	-	-	NO, -	-	-
Melanocytic nevus	YES, 12	January (19.71)	June (-9.45)	NO, -	-	-	NO, -	-	-
Melasma	NO, -	-	-	YES, 12	January (7.51)	June (-3.25)	NO, -	-	-
Nevus	NO, -	-	-	NO, -	-	-	NO, -	-	-
Nodule	YES, 12	April (6.94)	January (-5.74)	YES, 12	September (2.23)	December (-2.22)	NO, -	-	-
Papilloma	NO, -	-	-	NO, -	-	-	NO, -	-	-
Papule	NO, -	-	-	NO, -	-	-	NO, -	-	-
Perspiration	YES, 12	February (9.59)	June (-5.36)	YES, 12	March (8.35)	July (-9.27)	YES, 12	February (6.35)	June (-5.99)
Petechia	NO, -	-	-	NO, -	-	-	NO, -	-	-
Pustule	NO, -	-	-	NO, -	-	-	NO, -	-	-
Scar	NO, -	-	-	NO, -	-	-	NO, -	-	-
Skin fissure	NO, -	-	-	NO, -	-	-	NO, -	-	-
Skin rash	YES, 12	January (9.15)	May (-5.97)	NO, -	-	-	YES, 12	December (6.40)	June (-8.79)
Skin tag	YES, 12	January (10.85)	July (-9.02)	NO, -	-	-	NO, -	-	-
Skin ulcer	NO, -	-	-	NO, -	-	-	NO, -	-	-
Stretch marks	YES, 12	January (14.32)	May (-10.26)	YES, 12	January (14.27)	June (-11.58)	NO, -	-	-
Telangiectasia	YES, 12	January (9.97)	June (-13.74)	YES, 12	October (5.54)	July (-6.58)	YES, 12	January (5.00)	July (-3.58)
Vesicle	YES, 12	June (13.31)	January (-15.53)	YES, 12	April (9.11)	February (-13.95)	NO, -	-	-

Wart	YES, 12	January (14.07)	June (-7.22)	YES, 12	January (9.12)	June (-6.13)	NO, -	-	-
Xeroderma	YES, 12	July (9.10)	February (-10.49)	NO, -	-	-	NO, -	-	-

Table S6

Sensitivity analysis. Popularity of topics representing dermatologic diseases and non-medical in proportion to “Scar” (adjusted data; Relative Search Volume [RSV] over time).

No	Topic	Proportion of RSV to Scar
1.	Car	85.93
2.	Rome	8.04
3.	Star Wars	4.05
4.	Tomato	3.87
5.	FC Bayern Munich	1.55
6.	Scar	1.00
7.	Psoriasis	0.67
8.	Atopic dermatitis	0.54
9.	Scabies	0.49
10.	Melanoma	0.36
11.	Rosacea	0.25
12.	Basal-cell carcinoma	0.11
13.	Squamous cell skin cancer	0.01

Table S7

Sensitivity analysis. Time series analysis of non-adjusted topics representing skin diseases and non-medical topics.

Topic	Seasonal Mann-Kendall test	Slope [RSV/year]	TBATS (seasonality present, period [month])	Month with the highest seasonal component [RSV]	Month with the lowest seasonal component [RSV]	Seasonal component amplitude [RSV]
Skin diseases						
Atopic dermatitis	tau = 0.5; ***	1.06; ***	YES, 12	April (6.94)	September (-6.56)	13.5
Basal-cell carcinoma	tau = 0.15; **	0.26; 0.05	YES, 12	July (5.00)	December (-9.61)	14.62
Melanoma	tau = -0.22; ***	-0.79; ***	YES, 12	May (12.36)	December (-11.38)	23.74
Psoriasis	tau = 0.46; ***	0.95; ***	YES, 12	April (3.68)	December (-5.91)	9.59
Rosacea	tau = 0.23; ***	0.24; 0.162	YES, 12	April (14.62)	December (-9.48)	24.1
Scabies	tau = 0.77; ***	2.43; ***	YES, 12	October (3.51)	June (-2.51)	6.02
Squamous cell skin cancer	tau = 0.06; 0.246	-	YES, 12	June (7.43)	December (-6.79)	14.22
Non-medical						
Car	tau = -0.46; ***	-0.69; ***	YES, 12	July (4.44)	December (-4.92)	9.36
FC Bayern Munich	tau = 0.49; ***	1.51; ***	YES, 12	April (28.4)	June (-10.96)	39.36
Rome	tau = -0.50; ***	-1.09; ***	YES, 12	April (5.81)	August (-14.08)	19.89
Star Wars	tau = -0.36; ***	-0.30; 0.059	YES, 12	December (7.00)	August (-2.82)	9.81
Tomato	tau = 0.95; ***	3.40; ***	YES, 12	August (16.44)	November (-9.40)	25.84

*** $p < 0.001$; ** $p < 0.01$

Figure S2
Sensitivity analysis. Relative search volume over time of topics related to skin diseases and non-medical topics. Non-adjusted data.

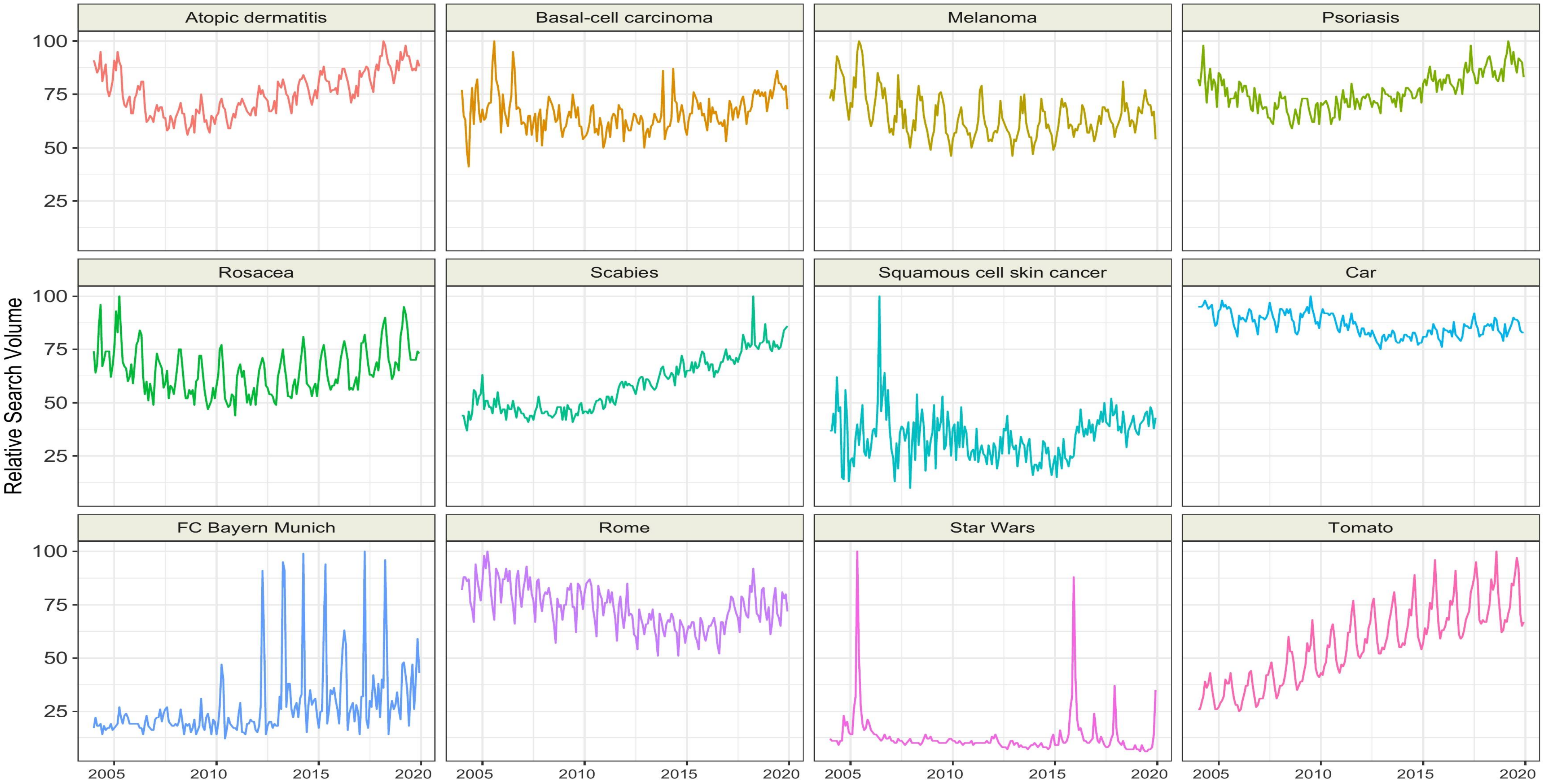

Supplement: Supplementary file 1 [file ijerph-18-02541-s001.pdf]
